# Responses to pandemic ASO3-adjuvanted A/California/07/09 H1N1 influenza vaccine in human immunodeficiency virus-infected individuals

**DOI:** 10.1186/1471-2172-13-49

**Published:** 2012-08-31

**Authors:** Deborah Kelly, Kimberley Burt, Bayan Missaghi, Lisa Barrett, Yoav Keynan, Keith Fowke, Michael Grant

**Affiliations:** 1 School of Pharmacy, Memorial University of Newfoundland and Labrador, NL, St. John’s, Canada; 2 Eastern Health Corporation of Newfoundland and Labrador, St. John’s, Canada; 3 Division of Infectious Diseases, Memorial University of Newfoundland and Labrador, NL, St. John’s, Canada; 4 National Institutes of Health Institute of Allergy and Infectious Diseases, Bethesda, MD, USA; 5 Departments of Medical Microbiology and Community Health Sciences, University of Manitoba, Winnipeg, MB, Canada; 6 Department of Medical Microbiology, University of Nairobi, Nairobi, Kenya; 7 Division of BioMedical Sciences, Faculty of Medicine, Memorial University of Newfoundland and Labrador, St. John’s, NL, Canada; 8 H1803- Immunology, Faculty of Medicine, Memorial University of Newfoundland, 300 Prince Philip Drive, St. John’s, NL, A1B 3V6, Canada

**Keywords:** HIV, influenza, pandemic, A/California/07/2009 H1N1 HA antigen, AS03 oil in water adjuvant, inflammation, CD4^+^ T cells, age

## Abstract

**Background:**

Influenza infection may be more serious in human immunodeficiency virus (HIV)-infected individuals, therefore, vaccination against seasonal and pandemic strains is highly advised. Seasonal influenza vaccines have had no significant negative effects in well controlled HIV infection, but the impact of adjuvanted pandemic A/California/07/2009 H1N1 influenza hemaglutinin (HA) vaccine, which was used for the first time in the Canadian population as an authorized vaccine in autumn 2009, has not been extensively studied.

**Objective:**

Assess vaccine-related effects on CD4^+^ T cell counts and humoral responses to the vaccine in individuals attending the Newfoundland and Labrador Provincial HIV clinic.

**Methods:**

A single dose of Arepanrix^TM^ split vaccine including 3.75 μg A/California/07/2009 H1N1 HA antigen and ASO3 adjuvant was administered to 81 HIV-infected individuals by intramuscular injection. Plasma samples from shortly before, and 1–5 months after vaccination were collected from 80/81 individuals to assess humoral anti-H1N1 HA responses using a sensitive microbead-based array assay. Data on CD4^+^ T cell counts, plasma viral load, antiretroviral therapy and patient age were collected from clinical records of 81 individuals.

**Results:**

Overall, 36/80 responded to vaccination either by seroconversion to H1N1 HA or with a clear increase in anti-H1N1 HA antibody levels. Approximately 1/3 (28/80) had pre-existing anti-H1N1 HA antibodies and were more likely to respond to vaccination (22/28). Responders had higher baseline CD4^+^ T cell counts and responders without pre-existing antibodies against H1N1 HA were younger than either non-responders or responders with pre-existing antibodies. Compared to changes in their CD4^+^ T cell counts observed over a similar time period one year later, vaccine recipients displayed a minor, transient fall in CD4^+^ T cell numbers, which was greater amongst responders.

**Conclusions:**

We observed low response rates to the 2009 pandemic influenza vaccine among HIV-infected individuals without pre-existing antibodies against H1N1 HA and a minor transient fall in CD4^+^ T cell numbers, which was accentuated in responders. A single injection of the Arepanrix^TM^ pandemic A/California/07/2009 H1N1 HA split vaccine may be insufficient to induce protective immunity in HIV-infected individuals without pre-existing anti-H1N1 HA responses.

## Background

In autumn 2009, the A/California/07/2009 H1N1 pandemic influenza vaccine was recommended as a priority to essential workers and high risk individuals, including individuals infected with human immunodeficiency virus (HIV) [1]. While previous studies of influenza vaccination in HIV-infected individuals showed no significant negative effects in the setting of effective highly active antiretroviral therapy (HAART) [2], the 2009 H1N1 influenza A vaccination campaign was the first to use the ASO3 adjuvant in the Canadian population [1]. The superiority of this oil in water adjuvant formulation of an influenza vaccine over non-adjuvanted formulations in enhancing vaccine antigen immunogenicity reduces the amount of H1N1 hemaglutinin (HA) antigen required per vaccine dose [3]. Therefore, it was recommended for use by Health Canada in order to optimize vaccine efficacy and distribution in the Canadian population [4]. Immunogenicity of vaccine antigens is enhanced by inflammation [5-7] and clinical trial data indicates an increased incidence of non-severe vaccine-associated adverse events following administration of the ASO3 adjuvanted A/California/07/2009 H1N1 vaccine (Arepanrix^TM^) compared to non-adjuvanted influenza antigen formulations [1,3,7]. These reported events were mainly symptoms of localized and systemic inflammation. Frequency and severity of adverse events reported was similar for HIV-infected and uninfected individuals [8,9]. Acute systemic inflammation with leukocytosis and impaired endothelial cell function was observed in a group of HIV-infected individuals monitored after receiving a similar A/California/07/2009 H1N1 vaccine formulation [10]. Thus, there is a high likelihood that this pandemic vaccine formulation induced stronger and/or more sustained inflammatory responses in the recipient population than those induced by previous seasonal influenza vaccine formulations. To obtain more information on factors affecting immunogenicity of Arepanrix^TM^ in the HIV-infected population, we performed a retrospective analysis of the vaccine responses of 80/81 vaccinated individuals attending the provincial HIV clinic in Newfoundland and Labrador, Canada. We used a sensitive microbead-based array assay to measure humoral responses and investigated baseline factors associated with vaccine immunogenicity. In addition, since inflammation and immune activation are risk factors for HIV disease progression, even in the setting of HAART [11,12], we assessed whether primary vaccination with Arepanrix^TM^ had any adverse effect on CD4^+^ T cell numbers in this group of 81 vaccinated individuals.

## Results

### Changes in CD4^+^ T lymphocyte counts in vaccine recipients

**Figure 1:**
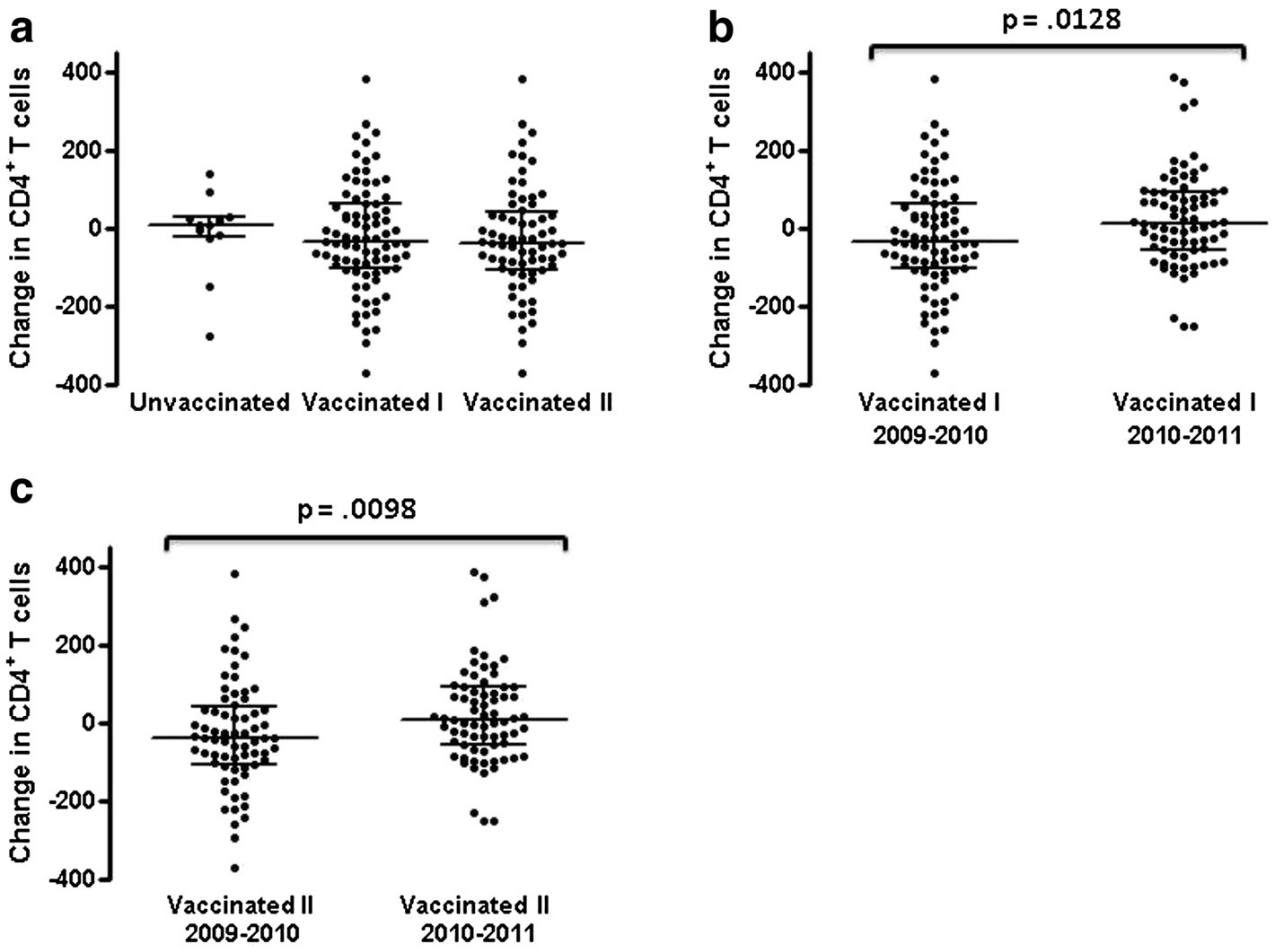
**Changes in CD4**^**+**^**T lymphocyte counts in vaccine recipients and non-recipients.** Differences in CD4^+^ T cell counts between the nearest sampling time point before vaccination and a second time point within 1–5 months after vaccination are shown for the vaccine recipients. The groups labeled “Vaccinated I” include all individuals receiving the vaccine while those labeled “Vaccinated II” exclude individuals whose HIV virus load changed by > 1 log_10_ over the observation period. Changes in CD4^+^ T lymphocyte counts over an equivalent period are shown for the group of non-vaccinated individuals (**a**). Changes in CD4^+^ T cells for the same group (I) and subgroup (II) of vaccine recipients over a similar time interval approximately 1 year later are compared in panels **b** and **c** to changes in CD4^+^ T cells observed over the post H1N1 vaccine period. Lines within groups show the group median with IQR and the p value indicating probability of no significant difference between groups is shown above lines spanning the groups being compared.

General characteristics of 93 HIV-infected individuals, of whom 81 received and 12 declined the 2009 H1N1 vaccine, are shown in Table 1. Of the vaccinated individuals, 63% (51/81) had plasma HIV viral loads below detectable levels (<1.6 log_10_), as did 67% (8/12) of the unvaccinated group. Twelve vaccinated individuals were not receiving antiretroviral therapy at the time of vaccination and through the follow-up period. For the 81 vaccine recipients, the mean peripheral blood CD4^+^ T cell count ± SD pre-vaccination was 534 ± 277/μL and post-vaccination was 500 ± 266/μL. Excluding those vaccinated individuals whose viral load changed by > 1 log_10_ over the observation period (12/81), the mean peripheral blood CD4^+^ T cell count ± SD pre-vaccination was 576 ± 268/μL pre-vaccination and 532 ± 261/μL post-vaccination. Neither change was statistically significant (p = 0.16 and p = 0.11, Mann–Whitney test for the entire group or with exclusion of those whose viral load changed by > 1 log_10_ over the observation period respectively). Over a similar time period, the mean CD4^+^ T cell count of the 12 unvaccinated HIV-infected individuals (none of whom had a change in HIV plasma viral load of > 1 log_10_ over the observation period) was 429 ± 235/μL initially and 418 ± 180/μL at the end of the observation period. There was no significant difference in the median change in CD4^+^ T cell counts between these groups over the autumn 2009 through early winter 2010 observation period whether the vaccinated individuals with a change in HIV plasma virus load of > 1 log_10_ were included (−34, IQR −104-65) or excluded (−39, IQR −105-42) versus 10, IQR −21-28, (Figure 1a). The small size of the group declining the vaccine (n = 12) precluded robust comparison of changes in CD4^+^ T cell numbers in inherently variable HIV-infected populations. Therefore, we also compared median change in circulating CD4^+^ T cell numbers within the vaccinated group over the period 1–5 months from receiving the vaccine to median change in CD4^+^ T cell numbers over an equivalent time period approximately 1 year later. While the seasonal flu vaccine in 2010 included A/California/07/09 H1N1, the ASO3 adjuvant was not used. Over the same time interval one year post A/California/07/09 H1N1 vaccination (2010/2011), the mean CD4^+^ T cell count of the H1N1-vaccinated group was 549 ± 283/μL initially and 573 ± 278/μL at the end of the observation period. There was no significant change in CD4^+^ T cell counts across this period (p = 0.3144, Mann–Whitney test). Excluding 4 cases where viral load rose or fell by > 1 log_10_ over the observation period, the mean CD4^+^ T cell count of the H1N1-vaccinated group was stable at 584 ± 259/μL initially and 593 ± 259/μL at the end of the observation period. The median change in CD4^+^ T cell numbers over a 1–5 month interval spanning fall/winter 2010/2011 was significantly different from the median change in CD4^+^ T cell counts that occurred in the 1–5 month (2009/2010) immediate post A/California/07/09 H1N1 vaccination period. This was consistent whether the 12 cases in 2009/2010 and 4 cases in 2010/2011 where viral load rose or fell by > 1 log_10_ were included (−34, IQR −104-65 versus 15, IQR −56-92, p = 0.0128, Mann–Whitney test, Figure 1b) or excluded from analysis (−39, IQR −105-42 versus 7.0, IQR −57-92, p = 0.0098, Mann–Whitney test, Figure 1c). These data suggest that primary A/California/07/09 H1N1 Arepanrix^TM^ vaccination had a slight and transient negative effect on circulating CD4^+^ T lymphocyte numbers in the group of HIV-infected individuals studied. However, the effect was relatively minor compared to the overall variation in CD4^+^ T cell counts observed over the follow-up period. Therefore, after measuring vaccine responses, we carried out a sub-analysis within the vaccinated group comparing the median change in CD4^+^ T cell numbers of responders to the median change in CD4^+^ T cell numbers of non-responders over the period 1–5 months from receiving the vaccine.

**Table 1:** General characteristics of H1N1 vaccine recipients and controls

	**Unvaccinated (n = 12)**	**Vaccinated (n = 81)**	**Responders (n = 36)**	**Non-responders (n = 44)**
^a^CD4^+^ T cells/μL Mean ± SD	420 ± 235	529 ± 269	609 ± 277	475 ± 241
0–200	1 (8.3%)	9 (11%)	3 (8.3%)	5 (11%)
201–350	5 (42%)	13 (16%)	4 (11%)	9 (20%)
351–500	2 (17%)	15 (19%)	4 (11%)	11 (25%)
>500	4 (33%)	44 (54%)	25 (69%)	19 (43%)
Log_10_ viral load Median (IQR)	1.60 (1.60-2.09)	1.60 (1.60-2.70)	1.60 (1.60-2.34)	1.60 (1.60-3.07)
≤1.60	8 (67%)	50 (62%)	23 (64%)	27 (61%)
1.61-2.60	3 (25%)	11 (14%)	7 (19%)	4 (9.1%)
2.61-3.00	0	2 (2.5%)	1 (2.8%)	1 (2.3%)
3.01-4.00	0	6 (7.4%)	1 (2.8%)	5 (11%)
>4.00	1 (8.3%)	12 (14%)	4 (11%)	7 (16%)
Age (years) Mean ± SD	45.7 ± 6.6	45.2 ± 7.2	44.2 ± 7.5	46.2 ± 7.0
Male	8 (67%)	56 (69%)	27 (75%)	29 (66%)
Female	4 (33%)	25 (31%)	9 (25%)	16 (36%)
^b^Pre-existing anti-H1N1	ND	28 (35%)	22 (61%)	6 (14%)

^a^Number of CD4^+^ T cells/μL peripheral blood at time nearest to, but before H1N1 vaccination.

^b^Number of individuals in each group with antibodies against H1N1 HA before vaccination.

### Vaccine responses

**Figure 2:**
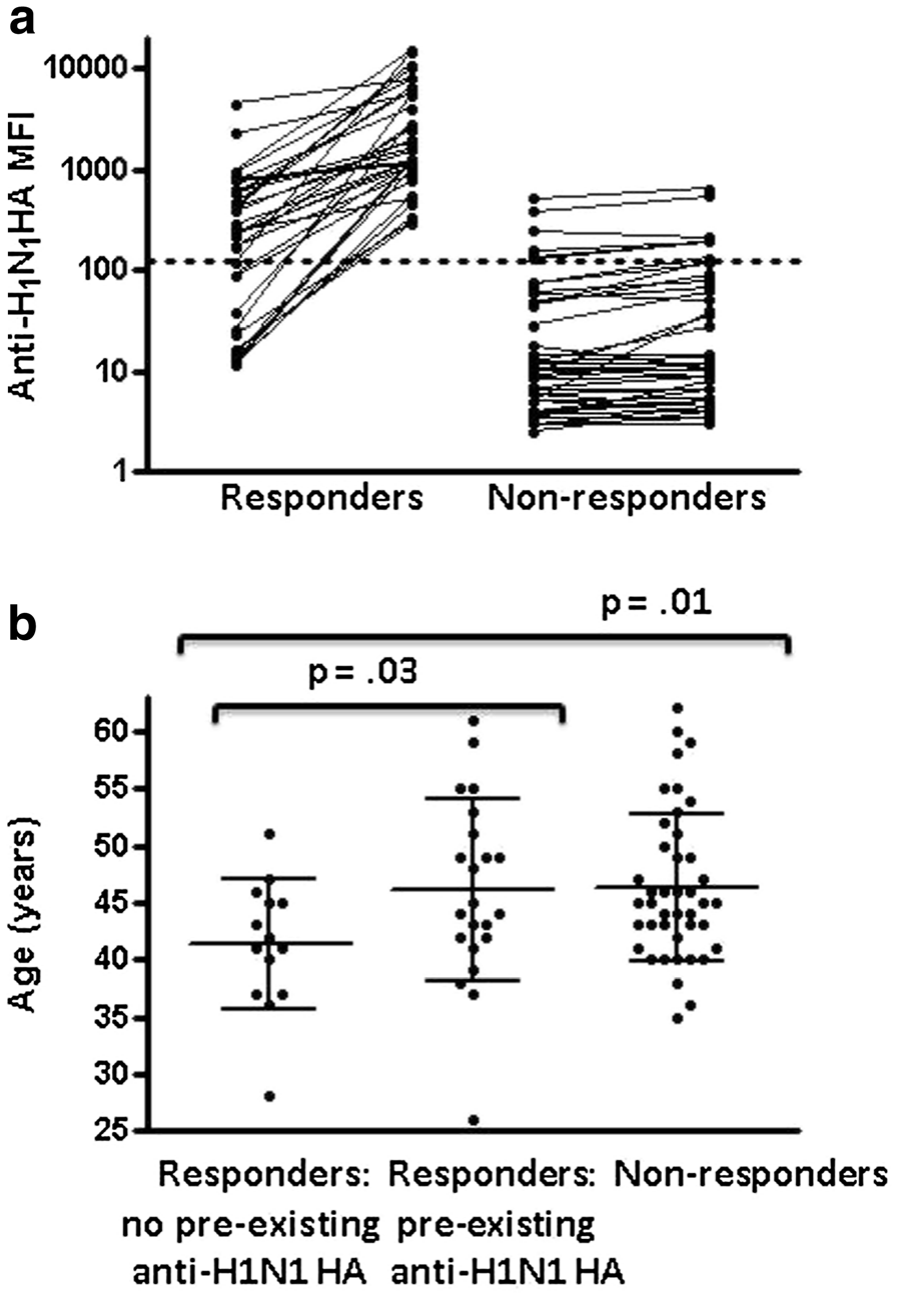
**Anti-H1N1 antibody levels detected by microbead-based array assay before and within 1–5 months after receiving the ASO3-adjuvanted A/California/07/09 H1N1 influenza HA vaccine for 80 HIV-infected individuals (a).** The dotted line at MFI 125 separates individuals with pre-existing anti-H1N1 antibodies from those without. Comparison of the age distribution for vaccinated individuals categorized as non-responders, responders or responders with pre-existing antibodies against A/California/07/09 H1N1 HA (**b**). Lines within groups show the group median with IQR and the p value indicating probability of no significant difference between groups is shown above lines spanning the groups being compared.

In cases where pre-existing antibodies reactive with A/California/07/09 H1N1 HA antigen were present [baseline mean fluorescence intensity (MFI) ≥ 125], an increase of ≥ 250 MFI units was considered a positive response. For those individuals without pre-existing antibody responses against A/California/07/09 H1N1 HA, an increase in MFI to ≥ 250 was considered a positive vaccine response. Consistent results were obtained with repeat testing of 80 pre- and post-vaccination sample pairs. Applying the criteria stated above, 28/80 individuals had pre-existing antibodies reactive with A/California/07/09 H1N1 HA antigen, 36/80 (45%, 95% CI 34%-56%) HIV-infected individuals responded to the vaccine and 44/80 were non-responders (Figure 2a). Twenty-two of the 28 (79%, 95% CI 63%-94%) HIV-infected individuals with pre-existing antibodies against A/California/07/09 H1N1 HA antigen responded to the vaccine compared to only 14 of the 52 (27%, 95% CI 15%-39%) without pre-existing antibodies against A/California/07/09 H1N1 HA antigen. Thus, HIV-infected individuals with pre-existing antibodies against A/California/07/09 H1N1 HA antigen were considerably more likely to respond to the vaccine (p = 0.00001, Fisher’s exact test). If some of those individuals with pre-existing antibodies were actually infected with A/California/07/09 H1N1 HA antigen over the follow-up period for plasma collection, this could result in overestimation of the response rate in this group. A limitation in the retrospective design of our study was the variable timing in collection of post-vaccination plasma samples for analysis of anti- A/California/07/09 H1N1 HA antibody responses.

### Comparison of responders and non-responders

**Figure 3:**
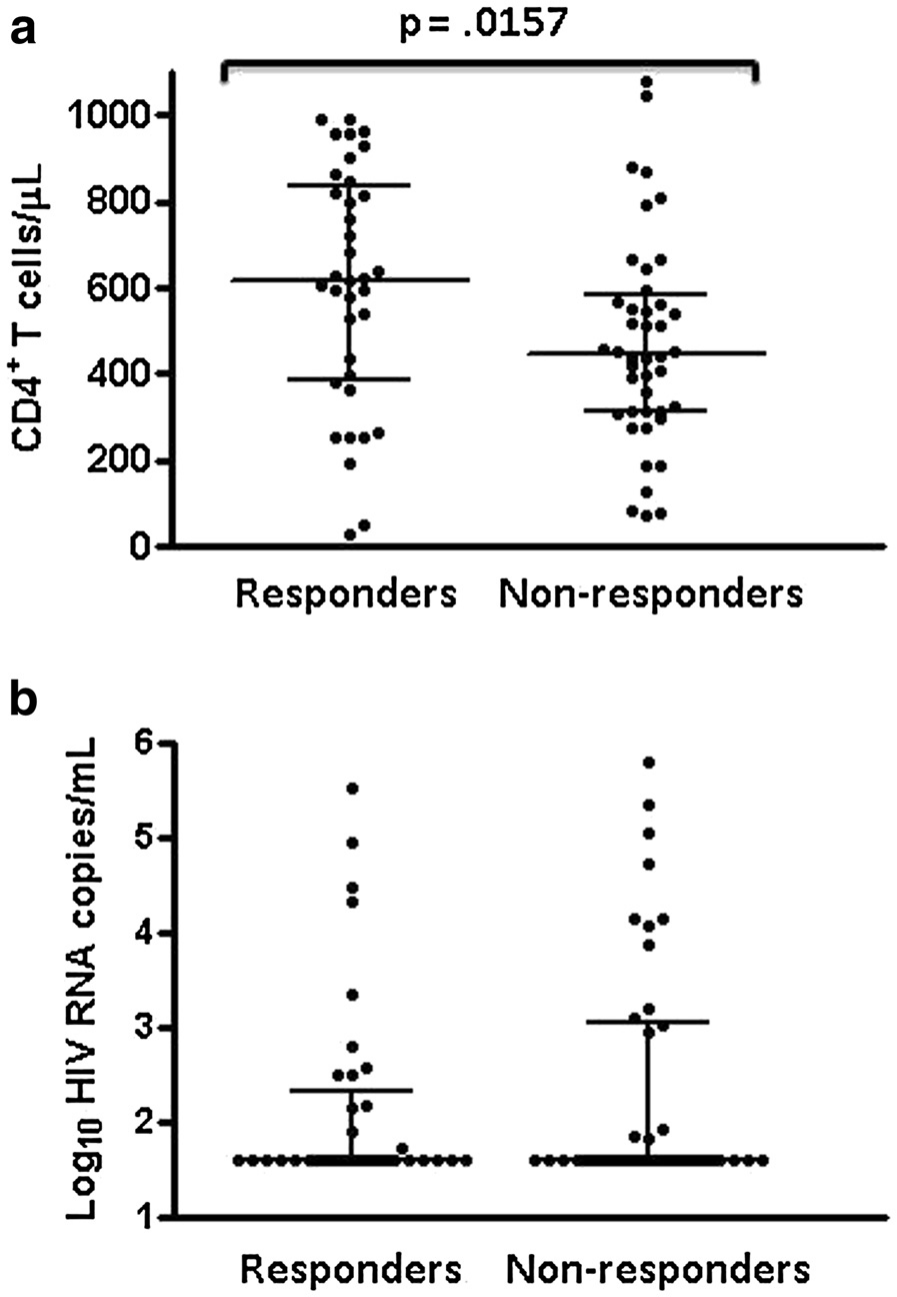
**Comparison of (a) CD4**^**+**^**T cell counts and (b) HIV viral loads between HIV-infected responders and non-responders to the A/California/07/09 H1N1 vaccine.** Lines within groups show the group median with IQR. The p value indicating probability of no significant difference between groups is shown above lines spanning the groups being compared.

There was no significant difference in mean age between responder and non-responder groups. However, as shown in Figure 2b, responders without pre-existing antibodies against A/California/07/09 H1N1 HA antigen had a lower mean age (41.4 ± 5.7 years) than either responders with pre-existing antibodies (46.1 ± 8.0 years, p = .03, Student’s *t* test) or non-responders (46.2 ± 7.0 years, p = .01, Student’s *t* test). Although there was a wide range of CD4^+^ T cell counts in both groups, the median CD4^+^ T cell count was significantly higher in the responder group than in the non-responder group (618, IQR 389–835 versus 446, IQR 314–582, p = 0.0157, Mann–Whitney test, Figure 3a). There was no significant difference in median CD4^+^ T cell nadir between responders and non-responders (126, IQR 83–199 versus 143, IQR 61–202), nor between responders with or without pre-existing antibodies against A/California/07/09 H1N1 HA antigen (165, IQR 69–270 versus 210, IQR 63–314) respectively. Median plasma HIV viral load at the time of vaccination was not significantly different between groups and several responders had relatively high plasma levels of HIV (Figure 3b). The effect of multiple variables on vaccine response was assessed by stepwise logistic regression. Age, gender, time since immunization, pre- and post-immunization CD4^+^ T cell counts, pre- and post- HIV viral load, and CD4^+^ T cell nadir did not independently affect response to influenza vaccine. Pre-existing influenza antibody was the only predictor (F = 119.9; p=0.002) of vaccine response in both univariate and multivariate analyses.

**Figure 4:**
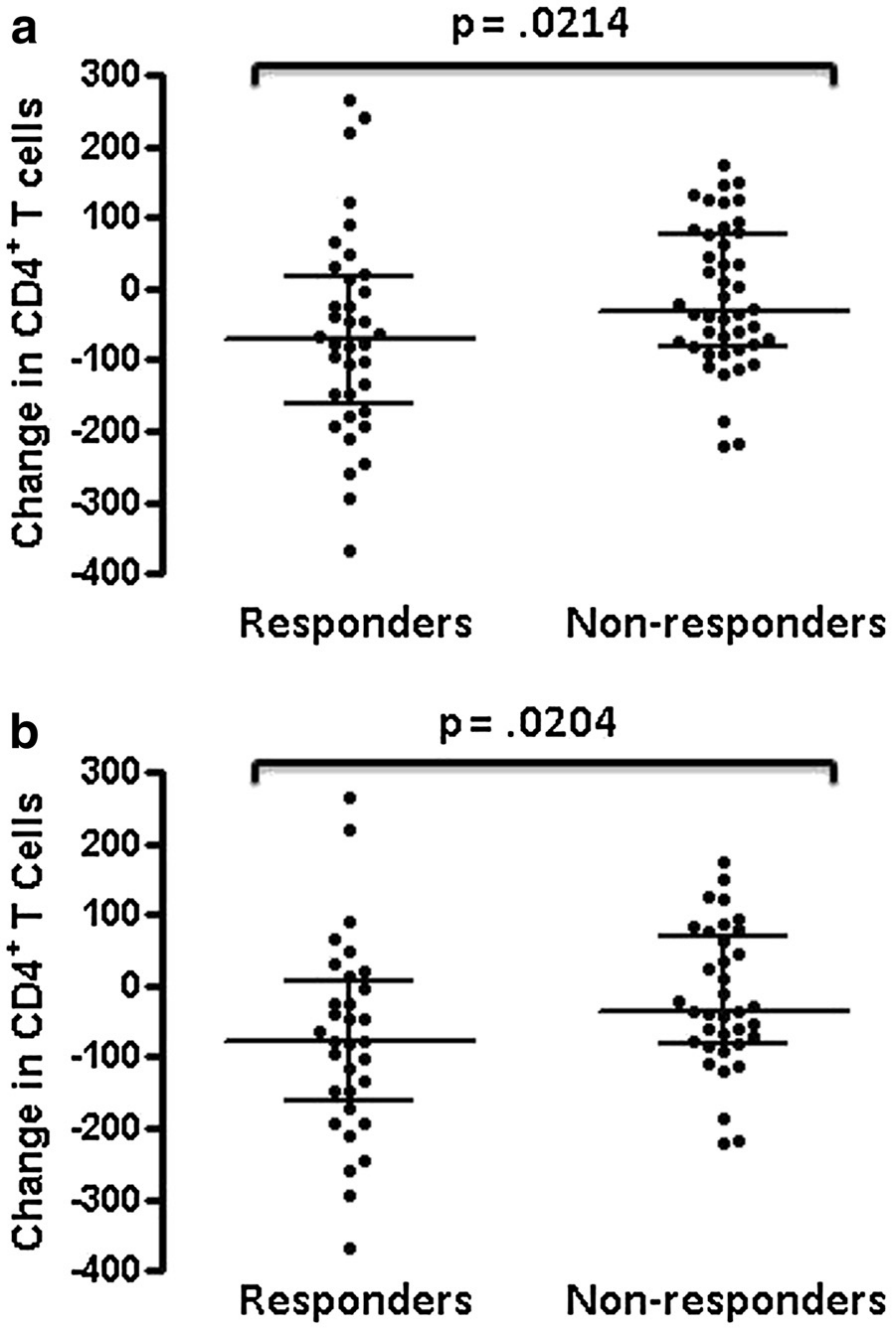
**Changes in CD4**^**+**^**T cell counts within 1 to 5 months of receiving the ASO3-adjuvanted A/California/07/09 H1N1 influenza HA vaccine for HIV-infected responders and non-responders (a).** In panel **b**, the changes in CD4^+^ T cell counts of individuals whose HIV virus load changed by > 1 log_10_ over the observation period were excluded from analysis. Lines within groups represent group median with IQR and the p value indicating probability of no significant difference between groups is shown above a line spanning the groups.

Although the median loss of CD4^+^ T cells over the period 1–5 months from receiving the vaccine was significantly greater than over the same time period 1 year later, the effect was small relative to overall variation in CD4^+^ T cell counts. To test for specificity of the effect in relation to the effect of vaccination, we compared CD4^+^ T cell losses between HIV-infected vaccine recipients who exhibited an increase in anti-A/California/07/09 H1N1 antibodies and those who did not. Our reasoning was that if CD4^+^ T lymphocyte counts were affected by the vaccine, the impact would be greatest in those making a measurable immune response against it. There was a significantly greater median loss of CD4^+^ T cells over the period from 1–5 months of receiving the A/California/07/09 H1N1 vaccine in the group of 36 individuals who responded to the vaccine compared to the 44 non-responders (−71, IQR −160-18 versus −31, IQR −80-78, p = 0.0214, Mann–Whitney test, Figure 4a). Results were similar when 4 responders and 8 non-responders whose HIV virus load changed by > 1 log_10_ over the observation period were excluded from analysis (−78, IQR −160-6.0 versus −36, IQR −80-70, p = 0.0204, Mann–Whitney test, Figure 4b).

## Discussion

There was broad uptake of the pandemic A/California/07/09 H1N1 HA influenza vaccine in autumn 2009 in both high risk and general populations. Including the ASO3 adjuvant in the formulation increased immunogenicity of the vaccine in the general population with no indication of severe adverse events, but there are few studies on the performance of the vaccine formulation in HIV-infected individuals. Using a sensitive microbead-based array assay [13], we observed a low response rate overall in HIV-infected individuals (45%, 95% CI 34%-56%), especially in those with little or no evidence of pre-existing antibodies against A/California/07/09 H1N1 HA (27%, 95% CI 15%-39%). In contrast, the response rate was considerably higher in those subjects with pre-existing antibodies (79%, 95% CI 63%-94%). Responders had a higher mean CD4^+^ T cell count than non-responders and responders without pre-existing antibodies against A/California/07/09 H1N1 HA and were younger than the non-responders and responders with pre-existing antibodies against A/California/07/09 H1N1 HA. Multivariate analysis did not indicate a difference in vaccine responsiveness, even when accounting for time since immunization.

These data indicate a predictable hierarchy of vaccine effectiveness in which youth, lesser evidence of HIV disease progression and previous sensitization all favour responsiveness. Previous studies of this vaccine formulation in HIV-infected individuals reported response rates of > 65% using standard HA inhibition assays [9,14-16]. Although our assay format may be more sensitive [13] and identify a higher percentage of subjects with pre-existing antibodies, the response rate we observed overall was still considerably lower. The study cohorts with > 65% vaccine response rates were similar in terms of age, concurrent CD4^+^ T cell counts and extent of viral suppression below detectable levels. Most subjects in our cohort were infected 15 or more years ago and thus, may have had lower CD4^+^ T cell nadirs than subjects in the other groups. Another factor potentially contributing to the disparity in observed response rates is the different times after vaccination that vaccine responses were measured. A different, but comparable MF59-adjuvanted H1N1 HA vaccine induced similar antibody responses in HIV-infected and control study groups, but responses declined rapidly between 1 and 6 months post-vaccination in the HIV-infected group [17]. Another study with the MF59-adjuvanted vaccine reported seroconversion rates of only 36% in the HIV-infected study group, based on a minimum four-fold increase over baseline titer [18]. These other studies used standards related to vaccine protection to assess seroconversion, which require development of an HA inhibition titer of ≥ 1/40 or a fourfold increase over baseline titer. In contrast, we relied exclusively on a sizable increase in anti-H1N1 Ab levels to indicate an immune response to the vaccine and to categorize recipients as responders or non-responders. As the microbead-based assay measuring MFI is more sensitive to changes in anti-H1N1 antibody levels than doubling dilution HA inhibition assays [13], this could influence a greater fraction of those with pre-existing anti-H1N1 Ab to be categorized as responders. If the response is subject to more rapid decay in non-sensitized HIV-infected individuals, then the longer time period we waited prior to assessment of responsiveness could have influenced a greater fraction overall to be categorized as non-responders. Obtaining samples systematically at earlier time points post vaccination could have changed our observed response rates. Overall, our results and others’ indicate that a prime-boost approach with this vaccine formulation might be more effective than a single administration at generating protective antibody levels in the HIV-infected population [19].

Another issue with use of this novel adjuvanted vaccine formulation in the HIV-infected population is the potential for adverse events associated with heightened or prolonged inflammation. While adverse events reported were primarily limited to localized pain, general fatigue and headache [1,3,8,9], we were struck by what appeared to be unusual declines in the CD4^+^ T cell counts of HIV-infected individuals within 1 to 5 months of receiving the vaccine. Therefore, we compared changes in CD4^+^ T cell counts between vaccine recipients and non-recipients, between vaccine responders and non-responders and among vaccine recipients over the post vaccine period and a similar time interval 1 year later. While the number of vaccine non-recipients in our study group was too low for robust comparisons, we did observe a slight, but significant fall in the CD4^+^ T cell numbers of vaccine recipients over the period following vaccination (November, 2009-March, 2010) compared to over a similar time period 1 year later. The fall in CD4^+^ T cell numbers over the time period following vaccination was significantly greater in the group of 36 vaccine responders compared to the group of 44 non-responders. In 2010, the seasonal flu vaccine did include A/California/07/2009 H1N1 HA, but was non-adjuvanted, suggesting the fall in CD4^+^ T cells observed post A/California/07/2009 H1N1 HA vaccination might reflect increased inflammation due to the adjuvant. If so, it may be informative to monitor CD4^+^ T cell counts at earlier time points post vaccination as it is somewhat surprising the effect persisted over the 1–5 months between pre and post-vaccination CD4^+^ T cell measurements. Again, our study was limited by its retrospective nature in that CD4^+^ T cell counts were not collected at completely systematic intervals relative to the timing of vaccination. Differences in CD4^+^ T cell changes over the 2009/2010 interval compared to 2010/2011 could also relate to the effects of primary versus booster vaccination with the inactivated A/California/07/2009 H1N1 HA antigen itself. A stronger effect might actually be anticipated upon secondary exposure, but with more rapid resolution. The effect on CD4^+^ T cells was slight relative to overall variation in CD4^+^ T cell numbers over the study period, raising some question as to its direct relationship to vaccination and biological significance. Given the potential impact of infection with influenza, the minor and transient effect on CD4^+^ T cell numbers from the vaccine itself should probably not be a consideration for HIV-infected individuals [20]. With the timing of plasma collection post vaccination, we cannot exclude the possibility that several individuals categorized as vaccine responders actually developed anti-A/California/07/2009 H1N1 antibodies as a result of infection. However, we feel it is more likely the observed accentuation of the effect in responders reflects the action of vaccine components affecting immunogenicity and reaffirms the general notion that immune activation can contribute to viral replication and the pathogenesis of CD4^+^ T cell loss in HIV infection [11,12,21]. Definition of operative mechanisms linking inflammation to CD4^+^ T cell loss in this light may be more relevant to the general pathogenesis of HIV infection than to vaccine-related adverse events.

## Conclusions

The response rate to the pandemic 2009 ASO3-adjuvanted A/California/07/2009 H1N1 vaccine Arepanrix^TM^ was 45%, 95% CI 34%-56% overall in a group of HIV-infected individuals followed in Newfoundland and Labrador and only 27%, 95% CI 15%-39% in those without pre-existing anti-H1N1 antibody responses. Either a series of injections, reformulation or an increase in antigen content may be necessary to achieve desired response levels in this population [19,22]. Vaccine administration was associated with a minor, transient loss of CD4^+^ T cells, which was greater in vaccine responders. The small magnitude and limited durability of the loss compared to overall variation in CD4^+^ T cell numbers and in relation to risks associated with influenza infection itself, suggest it should not be an issue in recommending vaccination against influenza in the HIV-infected population, even with adjuvanted vaccine formulations. However, the CD4^+^ T cell loss could be an important illustration of the role immune activation plays in the pathogenesis of HIV infection. As such, confirmation of the effect and research towards understanding the underlying mechanism(s) are desirable.

## Methods

### Subjects

This was a single centre retrospective study carried out in the provincial HIV clinic located in St. John’s, NL, Canada between October, 2009 and March, 2011. Ethical approval was received from the Health Research Ethics Authority of Newfoundland and Labrador. Individuals attending the St. John’s HIV clinic in autumn 2009 received priority access to the A/California/07/09 H1N1 vaccine Arepanrix^TM^ (GlaxoSmithKline). The clinic nurse directly administered the vaccine to 81 HIV-infected individuals attending the clinic during the first week of October 2009 (vaccinated group), while 12 other HIV-infected individuals under the clinic’s care declined vaccination; these individuals comprise the unvaccinated group. Plasma samples were collected with informed consent as part of an ongoing research study on immune responses in HIV infection. Pre-and post-vaccination plasma samples were obtained from 80/81 vaccinated individuals. Lymphocyte subset counts and plasma virus load (Roche Amplicor®) measurements for time points immediately prior to (“pre-vaccine period”) and between 1 and 5 months post vaccination (“post-vaccine period”) were collected from clinical chart information. Measurements were taken from corresponding time periods for the unvaccinated group for comparison purposes. Changes in CD4^+^ T cell counts from the pre-vaccine to post-vaccine period plasma samples were compared between the vaccinated and unvaccinated groups. In 12 cases where the plasma HIV viral load either rose or fell by more than 1 log_10_ across the pre and post vaccination span (4 responders and 8 non-responders), the changes in CD4^+^ T cell counts were excluded from comparison in sub-analyses, which were carried out separately. This was done because of the impact that large changes in HIV replication levels due to either de novo failure of, or response to antiretroviral therapy over the vaccine administration and follow-up period would likely have on CD4^+^ T cell numbers, irrespective of any vaccine effect. Comparison to changes in CD4^+^ T cell counts over a similar time period 1 year later was carried out within the vaccinated group using the same 1 log_10_ change in plasma HIV viral load exclusion criteria for sub-analysis.

### Anti-A/California/07/09 H1N1 hemaglutinin HA antibody measurements

Plasma samples from the pre-vaccine period and post-vaccine period were collected and stored at −80°C until tested. Antibody levels against the A/California/07/09 H1N1 HA antigen and 11 additional influenza HA antigens before and after vaccination were measured as in [13]. Briefly, recombinant HA antigens were coupled to 5.5 μm microspheres, which were then incubated with plasma samples. Antibodies binding to the beads were subsequently detected with biotinylated anti-human IgG antibodies and fluorochrome-conjugated avidin. The intensity of the anti-human IgG bound fluorochrome is proportional to the amount of antibody bound to A/California/07/09 HA, which is calculated by flow cytometry with calibrated standards. Where antibodies reactive with A/California/07/09 H1N1 HA antigen were present at baseline (MFI ≥ 125), increases of ≥ 250 MFI units were considered to indicate positive humoral responses to the vaccine. Without pre-existing antibody responses against A/California/07/09 H1N1 HA, increases in MFI to ≥ 250 were considered to indicate positive humoral responses to the vaccine.

### Statistical analysis

Data sets were assessed for normal distribution by the Kolmogorov-Smirnov, D’Agostini and Pearson and Shapiro-Wilk tests. If all three tests indicated a normal distribution, data was represented with mean ± standard deviation (SD) shown for the different groups and Student’s *t* test was used to compare means. If any of the data sets being compared did not meet test criteria for normal distribution, groups were compared by Mann–Whitney test. A one-tailed probability of less than 0.05 was considered significant. The difference in distribution of vaccine responses between groups with or without pre-existing anti-H1N1 antibodies was compared by Fisher’s exact test. Stepwise multiple logistic regression was performed using SPSS 19.0 statistical software to control for confounding variables.

## List of abbreviations

Ab: Antibody; HA: Hemaglutinin; HAART: Highly active antiretroviral therapy; HIV: Human immunodeficiency virus; IgG: Immunoglobulin G; IQR: Interquartile range; MFI: Mean fluorescence intensity; SD: Standard deviation; μg: Microgram; μL: Microliter.

## Competing interest

The authors declare that they have no competing interests.

## Authors’ contributions

DK conceived of the study, collected data, organized the involvement of different investigators and revised the manuscript. KB administered the vaccine, recorded vaccination dates and co-ordinated retrieval of CD4^+^ T cell counts and plasma virus loads for study subjects. BM was responsible for the clinical care of study subjects over part or all of the study period and revised the manuscript. LB contributed to the collection, analysis and interpretation of data and revised the manuscript. YK co-developed the bead-based anti-H1N1 antibody assay and revised the manuscript. KF co-ordinated measurement of anti-H1N1 antibody levels in pre- and post- vaccination plasma samples, collated the data and revised the manuscript. MG organized collection of the plasma samples, collated, analyzed and interpreted the data and drafted the manuscript. All authors read and approved the final manuscript.
